# Associations of Non-suicidal Self-Injury and Psychological Symptoms With Suicide Attempt in Adolescents: Are There Any Gender Differences?

**DOI:** 10.3389/fpsyt.2022.894218

**Published:** 2022-06-20

**Authors:** Huiqiong Xu, Rui Wang, Ruoyu Li, Zhengge Jin, Yuhui Wan, Fangbiao Tao

**Affiliations:** ^1^Department of Maternal, Child and Adolescent Health, School of Public Health, Anhui Medical University, Hefei, China; ^2^Department of Information Technology, Anqing Medical College, Anqing, China; ^3^Key Laboratory of Population Health Across Life Cycle (Anhui Medical University), Ministry of Education of the People's Republic of China, Hefei, China; ^4^NHC Key Laboratory of Study on Abnormal Gametes and Reproductive Tract, Hefei, China

**Keywords:** non-suicidal self-injury, psychological symptoms, suicide attempt, adolescents, gender

## Abstract

**Background:**

Psychological symptoms and non-suicidal self-injury (NSSI) are independently associated with suicide attempts (SA). Yet, no study has tested the interaction effects between NSSI and psychological symptoms on SA in community adolescent populations, or examined whether the interaction varies by gender. We sought to examine the interaction effects of NSSI and psychological symptoms on SA in adolescents and explore gender differences.

**Methods:**

A school-based health survey in 3 provinces in China was conducted between 2013–2014. 14,820 students aged 10–20 years completed standard questionnaires, to record the details of various psychological symptoms, SA and NSSI.

**Results:**

Psychological symptoms and NSSI were independently associated with a higher likelihood of SA in both boys and girls (*p* < 0.001). Adolescents with psychological, conduct or social adaptation symptoms without concurrent NSSI, were twice as likely to report SA (corresponding RORs were 1.80, 1.80 and 2.16, respectively; *p* < 0.01) than those who reported NSSI. Male adolescents with psychological, emotional, conduct or social adaptation symptoms had a higher risk of SA in the non-NSSI group than the NSSI group (corresponding RORs were 2.85, 2.26, 2.30 and 3.01 respectively; *p* < 0.01). While in girls, only adolescents with social adaptation symptoms had a higher risk of SA in the non-NSSI group than NSSI group (corresponding RORs was 1.71, *p* < 0.05). In the non-NSSI group, boys reporting psychological symptoms exhibited a higher likelihood of a SA than their female counterparts.

**Conclusion:**

Psychological symptoms and NSSI are independently associated with an increased risk of SA in adolescents. However, to some extent, NSSI may reduce the risk of SA among individuals with psychological symptoms, especially in boys.

## Introduction

Suicide is a major public health problem in adolescents as it constitutes the second leading cause of mortality in youth worldwide (1). It is estimated that for each death by suicide, an additional 25 suicide attempts (SA) are made, with the ratio even greater among teenage populations (2). Results from the 2017 national YRBSS (Youth Risk Behavior Surveillance System) in America have indicated that as much as 7.4% of students (10–24 years) had attempted suicide one or more times during the 12 months prior to the survey (3). Although the burden attributable to suicide has decreased in recent years, suicide continues to be the second principal cause of DALY (disability-adjusted life years) related to injury in the 15–24 years population within China (4). In addition, youth suicide attempt may increase risk for poor health and social functioning in adulthood, such as metabolic syndrome, elevated inflammation and long-term unemployment problems in adulthood (5). Therefore, the study of suicidal attempt and its related factors may contribute to the early detection and intervention strategies.

To date, an array of risk and protective factors have been examined in relation to youth suicide (6–10). Of which, the psychological symptoms and a history of non-suicidal self-injury (NSSI) being regularly cited as notable correlates in both cross sectional and prospective studies (11–15). For instance, one large cross sectional study of Chinese students, Tang and colleagues reported that the presence of NSSI was a commonly associated feature of SA irrespective of whether or not it was accompanied by suicidal ideation (12). Similarly, in a 8 year longitudinal study examining clinically depressed adolescents, Tuisku et al. concluded that NSSI remained a strong predictor of suicidal behavior over the course of the study (13). Likewise, the relationship between psychological symptoms and SA have been evidenced in both clinical and general population samples. A study of 17 622 students from 8 Chinese cities revealed that the prevalence of suicide ideation, plan and attempt were found to increase in line with a greater number of psychological symptoms (14, 15). Moreover, evidence from longitudinal studies suggest that the relationship between various psychiatric disorders and SA may actually strengthen over time (16).

A host of studies have also demonstrated a correlation between psychological symptoms and NSSI in adolescents (17, 18), with psychological symptoms frequently found to predict incident NSSI at follow-up (19). Such findings have led many researchers to conclude that NSSI may represent a form of maladaptive coping style which helps to modulate life stress and regulate affective and social experiences (20–22). This is supported by the DSM-5 definition of NSSI which suggests that a primary function of NSSI is to relieve a negative feeling or cognitive state and induce a positive emotional equilibrium (23). Nevertheless, there continues to be ambiguity around the function of self-harm in relation to psychological symptoms among different populations. Evidence from studies of patients with borderline personality disorders indicates that rather than resulting in emotional relief, NSSI appears to be associated with a further increase in negative emotion (24). It has also been suggested that when NSSI no longer effectively regulates increasingly stressful conditions individuals may start to engage in more extreme forms of self-injury (25). This fits with evidence from clinically depressed samples which demonstrates that the risk of suicidal behavior is elevated amongst adults with a history of NSSI and current Major Depressive Disorder (MDD) (26). Yet, it remains to be determined as to whether the affective regulation function of NSSI may show interim benefits, helping to attenuate the relationship between psychological symptoms and SA among general population adolescents.

Early evidence is conflicting in relation to the nature and underlying function of self-harm across genders with some studies suggesting that females more likely to use self-harm as a means of externalizing internal distress (27) while others have failed to support the existence of distinct gender differences (28). More recent evidence from You and colleagues indicates that while affect regulation remains one of the primary functions of NSSI for both genders, male adolescent self-injurers were more likely to endorse the social influence function than their female counterparts (20). Despite this, no study has explored gender differences in the interaction between NSSI and psychological symptoms on SA in general population adolescents.

The main hypothesis tested in this study is that individuals with psychological symptoms or NSSI would be associated with a higher risk of engaging in SA, and the interaction effects between psychological symptoms and NSSI increase the occurrence of SA, as well as there may be gender differences. Therefore, the aim of this paper is to investigate the independent effects of psychological symptoms and NSSI on SA in a sample of general population adolescents, secondly examine the interaction effects between psychological symptoms and NSSI on SA, and thirdly ascertain whether the interaction effects differs by gender.

## Methods

### Study Sample and Procedures

**Figure 1:**
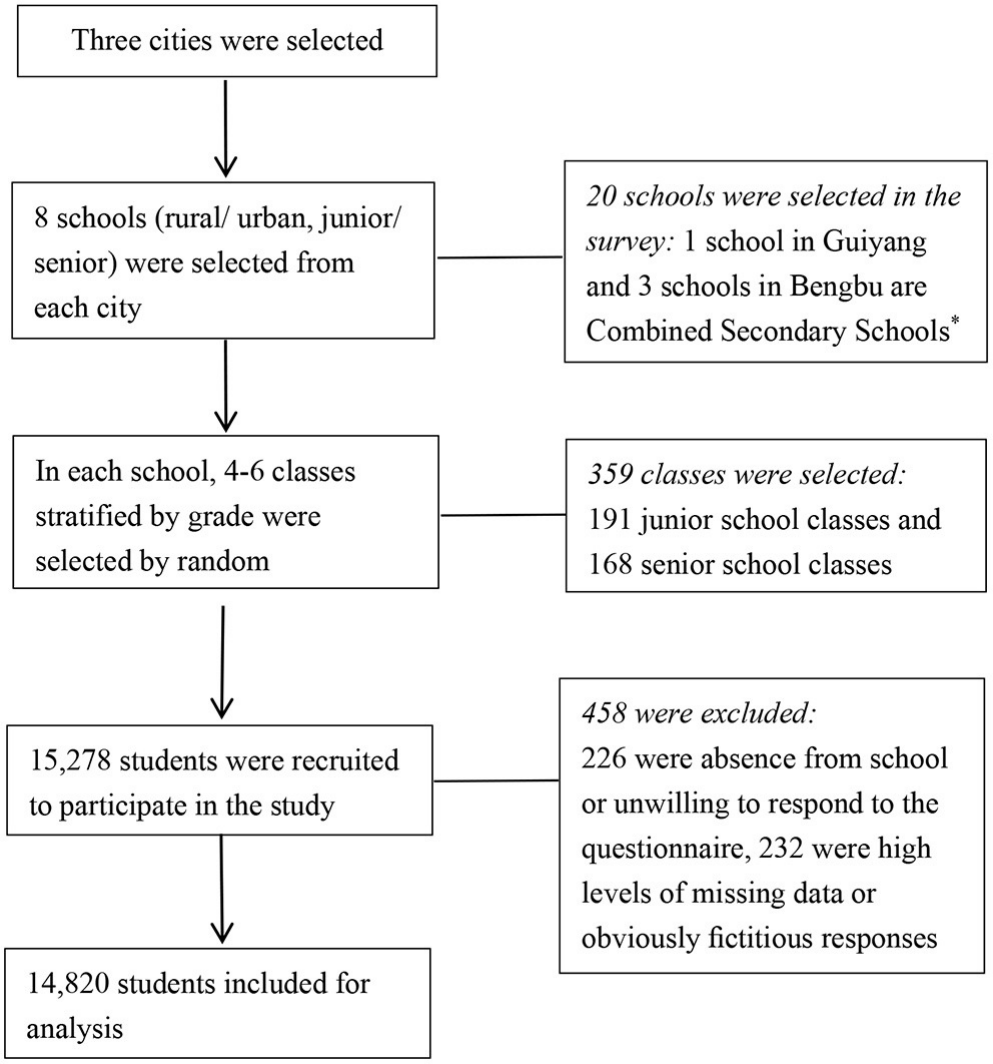
The flow diagram presenting the selection of study participants. ^*^Combined Secondary School refers to a school with both a junior school and a senior school.

The study population was derived from a health survey involving adolescents from junior and senior middle schools located in 3 areas (Bengbu in Anhui province, Zhengzhou in Henan province, Guiyang in Guizhou province) in China. The survey was conducted from November 2013 to January 2014. We have weighed the economic development and population composition of each region. These three provinces are broadly representative of the average level in China, and are also where our adolescent health research network is located, which facilitates data collection. Eight schools (four rural and four urban) were selected from each city, all of which were general junior and senior schools. As four schools consisted of junior and senior parts, the total number of the schools was 20 for the survey. A total of 15,278 students from grades 7–12 were selected to participate in the study and asked to complete an anonymous questionnaire. Of all participants, 458 (3.0%) were excluded from the study because of (1) absence from school on the day of the survey or unwillingness to respond to the questionnaire (*n* = 226), and (2) high levels of missing data or obviously fictitious or inconsistent responses (*n* = 232). Thus, a total sample of 14,820 participants were analyzed. And it has been reported in previous studies (29). The flow diagram was shown in Figure 1.

Before the questionnaire survey, informed consent was sought from parents/ guardians of each student. At the scene of investigation, the health survey team members explained the anonymous and confidential nature of the data to the students, and provided an opportunity for them to ask questions. If they were not willing to participate, they were allowed to withdraw from the study. The design and data collection procedures were approved by the Ethics Committee of Anhui Medical University (2012534). The research was performed in accordance with the Declaration of Helsinki.

## Measures

### Measurements of Sociodemographic and Adverse Childhood Experiences

#### Sociodemographic Profile

Demographic data for each participant was recorded, including age, gender, urban/rural residency, parents' education level (less than junior middle school, junior middle school, senior middle school, college or higher), self-perceived economic status of the family (poor, moderate, or good).

#### Adverse Childhood Experiences

The model adjusted for the presence of adverse childhood experiences (ACEs) due to the correlation with psychological symptoms, NSSI and SA. ACEs were defined as having experienced childhood maltreatment and/or household dysfunction. Childhood maltreatment was assessed using the Child Trauma Questionnaire (CTQ) (30), a widely used 28-item measure that examines five forms of childhood trauma (physical abuse, sexual abuse, emotional abuse, physical neglect and emotional neglect). Respondents were defined as “exposed to a category” if they responded “very often,” “often,” or “sometimes,” to any item in that category. Household dysfunction questions were derived from the Adverse childhood experiences international questionnaire (31). Respondents were defined as exposed to household dysfunction if they responded “Yes” to any item. In the present study, Cronbach's alpha coefficient for the ACEs scale was 0.726. Due to the high inter-relatedness of various types of ACEs (all *p* < 0.01), an ordinal 'number of ACEs types' score was created by summing the dichotomous ACEs items (range 0 to 6), and analyses were conducted with 4 categories of summed score (0, 1–2, 3–4, 5–6) (32).

### Measurements of Psychological Symptoms, NSSI and SA

#### Psychological Symptoms

Psychological symptoms in the past 12 months were examined by the psychological domain of the “Multidimensional Sub-health Questionnaire of Adolescents” (MSQA) (33, 34). Briefly, psychological symptoms were evaluated by 39 questions, consisting of 3 sub-scales: emotional symptoms (18 questions), conduct symptoms (8 questions) and social adaptation symptoms (13 questions). Emotional symptoms, included indicators of depression and anxiety, e.g., “Not enjoy anything at all.” Conduct symptoms, included paranoid and aggressive behaviors, e.g., “Feel like everyone's against you.” Social adaptation symptoms, included interpersonal difficulties such as poor school adjustment and forgoing social resources, e.g., “Feel uncomfortable in school life.” The internal consistency reliability coefficient of the emotional, conduct, social adaptation and psychological symptoms scale in the present study was 0.901, 0.818, 0.856, and 0.920 respectively. Each item contained 6 response options (none or lasting <1 week, lasting≥1 week, lasting≥2 weeks, lasting ≥1 month, lasting ≥2 months, or lasting≥3 months). Only the symptom duration lasting 1 month or more was assigned “yes (=1).” For each item, no symptoms or symptom durations of <1 month were assigned no“ (=0)”. Sub-scale scores and total scores were then calculated. In accordance with the national norm established for MSQA in China (34), the 90th percentile of national norm was selected as the cut-off points, which was 3, 1, 4, and 8 for emotional, conduct, social adaptation and psychological symptoms, respectively. Psychological and 3 subgroup symptoms were treated as dichotomous variables.

#### Non-suicidal Self-Injury

All participants received a screening question for non-suicidal self-injury(NSSI), which asked “In the past 12 months, have you ever harmed yourself in a way that was deliberate, but not intended to take your life? *Yes* or *No*?” A list of eight NSSI methods was then presented. The details of the questions were as follows: (1) hit yourself (2) pulled your own hair (3) banged your head or fist against something (4) pinched or scratched yourself (5) bitten yourself (6) cut or pierced yourself (7) burned yourself (8) Have you ever done some other things with the intention of hurting yourself (30). For those who confirmed that they had engaged in certain method of NSSI, the frequency was investigated, and the total frequency of NSSI was calculated. NSSI was treated as a dichotomous variable (frequency of NSSI [≥3], *Yes* or *No*) (35). The internal consistency reliability of NSSI was 0.749 in the current study.

#### Suicide Attempt

Suicide attempt (SA) was defined by responses to the question “Have you ever tried to kill yourself in the past 12 months?” (Yes/No).

### Statistical Analysis

Comparisons of sociodemographic risk factors, ACEs, psychological symptoms and SA between the NSSI and non-NSSI group were assessed using chi-squared test for categorical variables and one-way analysis of variance for continuous. Binomial logistic regression models were used to examine the associations between psychological symptoms and SA in the NSSI and non-NSSI groups. In the models, adjustment was made for age, gender, regional area, school, urban/rurality, mother's education level, economic status of the family and ACEs. In case collinearity problem, mother's educational level was adjusted for in the model, rather than father's educational level.

Gender differences in the associations between psychological symptoms and SA in the NSSI vs. non-NSSI were examined by calculating a ratio of two odds ratios (RORs) (36). All analyses were conducted with SPSS software, version 23.0 (SPSS Inc., Chicago, IL). A *p*-value of <0.05 was considered statistically significant in the analyses.

## Results

In 14,820participants, the mean age was 15.4 years (SD = 1.8), with a range of 10 to 20 years. 50.2% of the sample were females and 58.7% were recorded as living in a rural area. A total of 649(4.4%) adolescents reported that they had engaged in SA in the past 12 months. The rate of psychological symptoms and NSSI was 24.1% (3572) and 26.1% (3872), respectively.

Compared to the non-NSSI group, individuals with NSSI were more likely to report psychological symptoms, emotional symptoms, conduct symptoms, social adaptation symptoms and SA (*p* < 0.001). The details of differences between the NSSI and non-NSSI group can be seen in Table 1.

**Table 1:** Characteristics of participants by NSSI, *n*(%): 20 schools survey in China.

**Variables**	**Total**	**NSSI(Yes)**	**NSSI(No)**	***p*-value**
**Age (mean, SD)**	15.44 (1.8)	15.51(1.9)	15.41(1.8)	0.006
**Regional areas**				
Zhengzhou	5087(34.3)	1141(29.5)	3946(36.0)	<0.001
Guiyang	4617(31.2)	1385(35.7)	3232(29.6)	
Bengbu	5116(34.5)	1346(34.8)	3770(34.4)	
**Urban/rural**				
urban	6125(41.3)	1547(40.0)	4578(41.8)	0.043
rural	8695(58.7)	2325(60.0)	6370(58.2)	
**Father's education level**				
college or more	2230(15.0)	552(14.3)	1678(15.3)	0.002
senior middle school	3129(21.1)	808(20.9)	2321(21.2)	
junior middle school	6039(40.7)	1534(39.6)	4505(41.1)	
less than junior middle school	3422(23.1)	978(25.2)	2444(22.4)	
**Mother's education level**				
college or more	1706(11.5)	411(10.6)	1295(11.8)	<0.001
senior middle school	2826(19.1)	705(18.2)	2121(19.4)	
junior middle school	5369(36.2)	1350(34.9)	4019(36.7)	
less than junior middle school	4919(33.2)	1406(36.3)	3513(32.1)	
**Economic status of family**				
good	1841(12.4)	438(11.3)	1403(12.8)	<0.001
moderate	10306(69.6)	2542(65.7)	7764(70.9)	
poor	2673(18.0)	892(23.0)	1781(16.3)	
**ACEs score**				
0	1573(10.6)	169(4.4)	1404(12.8)	<0.001
1 2	6388(43.1)	1310(33.8)	5078(46.4)	
3 4	5303(35.8)	1679(43.4)	3624(33.1)	
5 6	1556(10.5)	714(18.4)	842(7.7)	
**Psychological symptoms**				
No	11248(75.9)	2139(55.2)	9109(83.2)	<0.001
Yes	3572(24.1)	1733(44.8)	1839(16.8)	
**Emotional symptoms**				
No	10514(70.9)	1886(48.7)	8628(78.8)	<0.001
Yes	4306(29.1)	1986(51.3)	2320(21.2)	
**Conduct symptoms**				
No	10488(70.8)	1891(48.8)	8597(78.5)	<0.001
Yes	4332(29.2)	1981(51.2)	2351(21.5)	
**Social adaptation symptoms**				
No	11588(78.2)	2330(60.2)	9258(84.6)	<0.001
Yes	3232(21.8)	1542(39.8)	1690(15.4)	
**Suicide attempt (SA)**				
No	14171(95.6)	3476(89.8)	10695(97.7)	<0.001
Yes	649(4.4)	396(10.2)	253(2.3)	

Psychological symptoms, including thee emotional, conduct and social adaptation subscales and NSSI were highly correlated in both males and females (*p* < 0.001). Psychological symptoms and NSSI had independent effects on SA after controlling for age, regional areas, school, urban/rurality, mother's education level, economic status of family and ACEs in males and females (shown as Supplementary Tables 1, 2).

**Figure 2:**
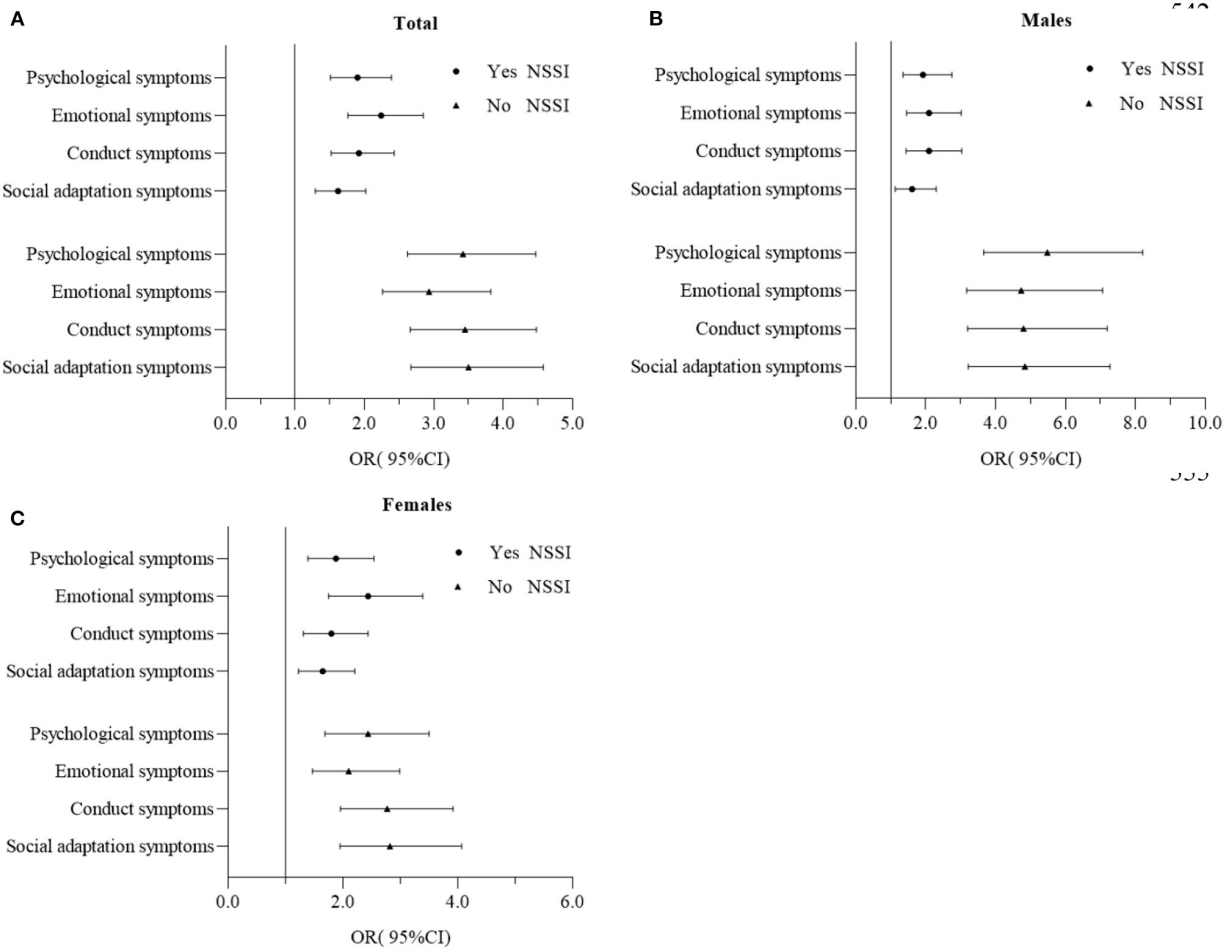
Associations of psychological symptoms with NSSI in **(A)** total sample **(B)** males and **(C)** females, OR(95%CI).

In the multivariate adjusted logistic regression models, as shown in Figure 2, Table 2, psychological symptoms were positively associated with SA in both NSSI groups (with or without NSSI), however in adolescents with psychological, conduct and social adaptation symptoms, the non-NSSI group were twice as likely to report SA (corresponding RORs were 1.80, 1.80 and 2.16, respectively; *p* < 0.01) than those with NSSI. There was an interaction effect between psychological symptoms, conduct symptoms, social adaptation symptoms and NSSI on SA in the total sample (corresponding OR(95%CI) were 0.56(0.40–0.79), 0.59(0.42–0.82) and 0.47(0.33–0.65), respectively; *p* < 0.01), but no interaction effect was found for emotional symptoms and NSSI on SA (OR=0.77, 95%CI: 0.54–1.09; *p* = 0.135).

**Table 2:** Number, % and adjusted OR of SA by psychological symptoms and NSSI.

**Group**		***n*(%)**	***OR*(95%*CI*)^a^**	***p*–Value**	**Ratio of two ORs in NSSI (No)**	
					**vs. NSSI (Yes)**	
					***ROR*(95%*CI*)**	*p*-Value ^*^
**Psychological symptoms**	*NSSI*					
No	No	140(1.5)	1.0			
Yes	No	113(6.1)	3.42(2.62–4.47)	<0.001		
No	Yes	147(6.9)	1.0			
Yes	Yes	249(14.4)	1.90(1.51–2.39)	<0.001	1.80(1.27-2.56)	0.001
**Emotional symptoms**	*NSSI*					
No	No	132(1.5)	1.0			
Yes	No	121(5.2)	2.93(2.26–3.82)	<0.001		
No	Yes	113(6.0)	1.0			
Yes	Yes	283(14.2)	2.24(1.76–2.85)	<0.001	1.31(0.92–1.87)	0.140
**Conduct symptoms**	*NSSI*					
No	No	120(1.4)	1.0			
Yes	No	133(5.7)	3.45(2.66–4.48)	<0.001		
No	Yes	121(6.4)	1.0			
Yes	Yes	275(13.9)	1.92(1.52–2.43)	<0.001	1.80(1.27–2.55)	0.001
**Social adaptation symptoms**	*NSSI*					
No	No	146(1.6)	1.0			
Yes	No	107(6.3)	3.50(2.67–4.58)	<0.001		
No	Yes	184(7.9)	1.0			
Yes	Yes	212(13.7)	1.62(1.29–2.02)	<0.001	2.16(1.52–3.07)	<0.001

*SA, suicidal attempt; NSS, non-suicidal self-injury*.

a *Adjusted for gender, age, regional areas, school, urban/rurality, mother's education level, economic status of family and ACEs*.

* *2-side P-value*.

Adjusted OR for SA by psychological symptoms and NSSI, in males and females are shown in Figure 2, Tables 3, 4. In males, adolescents with psychological, emotional, conduct and social adaptation symptoms had a higher likelihood of SA in the non-NSSI group than NSSI group (corresponding RORs were 2.85, 2.26, 2.30 and 3.01 respectively; *p* < 0.01). While in females, adolescents with social adaptation symptoms had a higher likelihood of SA in the non-NSSI group than NSSI group (corresponding RORs was 1.71, *p* < 0.05). There was an interaction effect of psychological symptoms, emotional symptoms, conduct symptoms, social adaptation symptoms and NSSI on SA in males (corresponding OR(95%CI) were 0.37(0.22–0.62), 0.45(0.27–0.76), 0.46(0.27–0.78) and 0.35(0.21–0.58), respectively; *p* < 0.01). While the only interaction effect was observed for social adaptation symptoms and NSSI on SA in females (OR = 0.56, 95%CI: (0.35–0.87); *p* = 0.011). In the non-NSSI group, males with symptoms of psychological, emotional and conduct problems were more likely to report SA than females (corresponding RORs were 2.25, 2.25 and 1.73 respectively; *p* < 0.05), while in the NSSI group, no gender differences were found.

**Table 3:** Number, % and adjusted OR of SA by psychological symptoms and NSSI in males.

**Group**		***n*(%)**	***OR*(95%*CI*)^a^**	***p–*Value**	**Ratio of two ORs in NSSI (No)**	
					**vs. NSSI (Yes)**	
					***ROR*(95%*CI*)**	*p*–Value ^*^
**Psychological symptoms**	*NSSI*					
No	No	52(1.2)	1.0			
Yes	No	56(7.0)	5.48(3.66–8.21)	<0.001		
No	Yes	62(5.1)	1.0			
Yes	Yes	89(10.6)	1.92(1.35–2.75)	<0.001	2.85(1.67–4.89)	<0.001
**Emotional symptoms**	*NSSI*					
No	No	49(1.1)	1.0			
Yes	No	59(5.8)	4.73(3.17–7.06)	<0.001		
No	Yes	53(4.7)	1.0			
Yes	Yes	98(10.4)	2.09(1.45–3.01)	<0.001	2.26(1.32–3.89)	0.003
**Conduct symptoms**	*NSSI*					
No	No	45(1.1)	1.0			
Yes	No	63(5.8)	4.80(3.20–7.19)	<0.001		
No	Yes	47(4.4)	1.0			
Yes	Yes	104(10.5)	2.09(1.44–3.03)	<0.001	2.30(1.33–3.98)	0.003
**Social adaptation symptoms**	*NSSI*					
No	No	56(1.2)	1.0			
Yes	No	52(6.5)	4.84(3.22–7.27)	<0.001		
No	Yes	74(5.7)	1.0			
Yes	Yes	77(10.1)	1.61(1.13–2.30)	0.009	3.01(1.75–5.16)	<0.001

*SA, suicidal attempt; NSSI, non–suicidal self–injury*.

a *Adjusted for age, regional areas, school, urban/rurality, mother's education level, economic status of family and ACEs*.

* *2–side P-value*.

**Table 4:** Number, % and adjusted OR of SA by psychological symptoms and NSSI in females.

**Group**		***n*(%)**	***OR*(95%*CI*)^a^**	***p–*Value**	**Ratio of two ORs in NSSI (No)**	
					**vs. NSSI (Yes)**	
					***ROR*(95%*CI*)**	*p*–Value ^*^
**Psychological symptoms**	*NSSI*					
No	No	88(1.9)	1.0			
Yes	No	57(5.5)	2.44(1.69–3.50)	<0.001		
No	Yes	85(9.3)	1.0			
Yes	Yes	160(17.9)	1.88(1.39–2.54)	<0.001	1.30(0.81–2.08)	0.280
**Emotional symptoms**	*NSSI*					
No	No	83(1.9)	1.0			
Yes	No	62(4.7)	2.10(1.47–2.99)	<0.001		
No	Yes	60(7.8)	1.0			
Yes	Yes	185(17.7)	2.44(1.75–3.39)	<0.001	0.86(0.53–1.40)	0.544
**Conduct symptoms**	*NSSI*					
No	No	75(1.7)	1.0			
Yes	No	70(5.5)	2.77(1.96–3.92)	<0.001		
No	Yes	74(9.0)	1.0			
Yes	Yes	171(17.3)	1.80(1.31–2.44)	<0.001	1.54(0.97–2.45)	0.070
**Social adaptation symptoms**	*NSSI*					
No	No	90(1.9)	1.0			
Yes	No	55(6.1)	2.82(1.95–4.07)	<0.001		
No	Yes	110(10.6)	1.0			
Yes	Yes	135(17.4)	1.65(1.23–2.21)	0.001	1.71(1.07–2.74)	0.026

*SA, suicidal attempt; NSSI, non–suicidal self–injury*.

a *Adjusted for age, regional areas, school, urban/rurality, mother's education level, economic status of family and ACEs*.

* *2–side P–value*.

## Discussion

The finding that psychological symptoms and NSSI represent independent risk factors for suicidal behaviors are akin to numerous prior studies (9–15). However, the current study advances the existing knowledge by demonstrating that in some instances, NSSI may actually weaken the strength of the relationship between psychological symptoms and SA in the short term. Unlike studies which have postulated that a the risk of suicide is heightened among individuals with a history of NSSI in conjunction with current clinical depression (26), the reverse effect was observed in the current study of general population adolescents whereby, in the absence of recent NSSI, adolescents with current psychological symptoms were twice as likely to report SA. By implication, this reinforces the assumption that NSSI may temporarily serve to regulate psychological distress. For instance, Hamza et al. found that the interaction between NSSI and individual's level of intrapersonal distress on suicidal behavior, and the association between NSSI and SA was stronger among individuals experiencing high levels of psychological distress (37). One possible interpretation for this finding is that in instances where NSSI fails to effectively regulate distress, individuals may turn to more extreme forms of self-injury which share similar experiential qualities, such as suicidal behaviors (25). This is in keeping with research which suggests that NSSI commonly serves an emotional regulation function among individuals experiencing internal distress resulting from depressive symptoms (22). Our results are consistent with the belief that NSSI may function as a maladaptive coping mechanism used to regulate affective or interpersonal difficulty (20, 23). This is also consistent with findings from Linda et al. who suggest that in the context of life stress, even passive problem coping weakened the relationship between life stress and suicidal ideation for individuals with a history of suicide attempts (38).

The results of the present suggest that the relationship between NSSI, psychological symptoms and risk of SA may manifest differently in males and females. Among those who did not self-harm but reported psychological symptoms, the risk of SA was higher for males than their female counterparts. Moreover, among those who reported self-harm, the risk of SA was reduced only among females with social adaption symptoms, whereas for males, the risk was reduced for those with multiple forms of psychological symptoms.These findings may in part, be explained by studies which have shown that females tend to make greater use of active coping skills and are more likely to seek social support than males (39, 40). It may be reasonable therefore to assume that, since males are less likely to implement active coping strategies and draw upon social support in times of psychological distress, NSSI may represent an alternative, albeit maladaptive, coping mechanism which can reduce the risk of SA in the interim.

Furthermore, the results provide substantive support for the divergent function of self-harm in males and females. In previous Chinese cohort studies, adolescent male self-injurers were found to be more likely than their female counterparts to endorse functions which included increasing control and getting others' attention (20). As the authors indicate, this suggests that the externalizing motivations may be a significant driver of self-harm amongst males while females are more likely to endorse internationalizing motivators. In cases where males exhibited psychological symptoms, NSSI may help them to better externalize their emotions and consequently, reduce the likelihood of engaging in SA. Therefore, the adaptive or maladaptive nature of NSSI may depend on the psychological symptoms and gender.

### Strengths and Limitation of the Study

The strengths of the current study include the following: First, we were able to examine the association between psychological symptoms, NSSI and SA among a large-scale school-based adolescent sample, covering urban and rural areas in China.Second, to our knowledge, the current study was the first study to identify the interaction effects between psychological symptoms and NSSI on SA. Third, the large sample, including many co-variables at data collection has provided enough statistical power to examine gender differences of the role.

However, several limitations should be considered when interpreting these results. First, it is difficult to establish a temporal order between psychological symptoms, NSSI and SA, due to the cross-sectional design. Moreover, the replication of these findings using longitudinal data would also assist in establishing the chronological stability of the relationships as it is possible that the persistent use of this maladaptive coping strategy this may serve to increase the long term risk. We need therefore to identify if this is the first instance of self-harm or whether it represents a history of self-harm behaviors as repeated use of this strategy may serve to habituate the individual to pain and fear of death and ultimately increase the risk of suicide. Second, due to the reliance on self-reported questionnaires it is possible that recall bias may exist and that rates of NSSI and SA maybe under-reported because of the sensitive nature of the questions. Future studies may therefore seek to integrate more robust measures of NSSI and psychological symptoms. Third, the extent to which these findings can be generalized to adolescents in other countries or cultures is also unclear as all participants in this study were located in mainland China. Fourth, this study can not get the cut-off for NSSI to regulate emotions, whether it is possible in the short term, or at low frequency, NSSI may be helpful to emotion regulation, it also needs to be further explored in the future. Fifth, there are many other risk factors for suicide behavior (41), including the population-level risk factors, such as social culture, economic level and media and Internet publicity, especially among teenagers, the Internet is an important way to seek suicide-related information and news (8). In addition, including the individual-level risk factors, such as the presence of suicidal behavior in family members, genetic factors of specific genes, and other psychosocial, demographic and biological factors also increase the susceptibility to suicide. Although age, residence, parents' education level, family economic status and ACEs were adjusted in this study, other important influencing factors of SA may be neglected, such as emotional temperament, one study have found that affective temperament-types were independently and more strongly associated with SA than was diagnosis of a major affective disorder in psychiatric inpatients (42). Therefore, these still need to be further studied in the sample of community adolescents. Finally, on the basis of trying to capture the impact of other influential factors, the interaction between these factors is further explored. Existing studies indicated that a variety of psychological and behavioral problems in adolescents often do not exist alone, but appear to be clustered and interrelated (43, 44).

### Implications

The findings indicate that psychological symptoms and NSSI are independently associated with an increased risk for SA in school adolescents, which by extension, would imply that the combination of these factors may be particularly detrimental in increasing the likelihood of behavioral enactment. However, to the contrary, the current results suggest that in adolescent males at least, NSSI may play a functional role the buffering impact on the relationship between psychological symptoms and SA. So far, from a clinical and developmental perspective, many theories and related studies have linked NSSI to mood disorders (21, 45, 46). Study focusing on the function of NSSI have shown that adolescents may use self-harm to reduce strong negative effects as well as avoid unnecessary emotions (21). These findings are broadly consistent with the NSSI view of developmental psychopathology, particularly with current theoretical concepts. For example, in the biosocial model of mood disorders (47, 48) adverse life events may cause individuals to suffer excessive stress, which in turn leads to self-harm, which is a maladaptive strategy to relieve pain.

With these in mind, intervention and prevention programs for SA may utilize these findings to identify higher-risk individuals early and tailor programs. Clinical assessment and interventions may benefit from an understanding that problem solving is especially important in responding to psychological symptoms among males, and moreover, that NSSI among this population may be helpful rather than maladaptive under these circumstances in the short term. From this, clinicians should assist these individuals choose more adaptive solutions to problems instead of NSSI, in order to weaken the impact of psychological symptoms on risk for SA.

## Conclusion

Psychological symptoms and NSSI are associated with an increased the risk of SA in adolescents however in some instances, NSSI may help to temper the relationship between psychological symptoms and SA, especially in males. Further research is needed to better understand the function of NSSI in individuals with psychological problems and how this might help individuals avoid transitioning toward suicidal behaviors particularly in males.

## Data Availability Statement

The raw data supporting the conclusions of this article will be made available by the authors, without undue reservation.

## Ethics Statement

The studies involving human participants were reviewed and approved by the Ethics Committee of Anhui Medical University (2012534). Written informed consent to participate in this study was provided by the participants' legal guardian/next of kin.

## Author Contributions

HX reviewed the topic related literature and drafted the first version of manuscript. RW involved in interpretation of the data and revision of the manuscript. YW and FT performed the study design, coordination, data collection, and the guarantors for the study. RL and ZJ worked on data analysis. All authors checked interpreted results and approved the final version.

## Funding

Funding for the project was provided by National Natural Science Foundation of China (81773453 and 81202223). The funders had no role in study design, data collection and analysis, decision to publish, or preparation of the manuscript.

## Conflict of Interest

The authors declare that the research was conducted in the absence of any commercial or financial relationships that could be construed as a potential conflict of interest.

## Publisher's Note

All claims expressed in this article are solely those of the authors and do not necessarily represent those of their affiliated organizations, or those of the publisher, the editors and the reviewers. Any product that may be evaluated in this article, or claim that may be made by its manufacturer, is not guaranteed or endorsed by the publisher.

## Abbreviations

NSSI: non-suicidal self-injury
SA: suicide attempts
RORs: ratio of two odds ratios
YRBSS: Youth Risk Behavior Surveillance System
DALY: disability-adjusted life years
DSM-5: Diagnostic and statistical manual of the American Psychiatric Association. 5th ed
MDD: Major Depressive Disorder
ACEs: adverse childhood experiences
CTQ: Child Trauma Questionnaire
MSQA: Multidimensional Sub-health Questionnaire of Adolescents
SPSS: Statistical Product and Service Solutions
SD: standard deviation
OR: odds ratio.
