# Supplementary material for: Associations of Non-suicidal Self-Injury and Psychological Symptoms With Suicide Attempt in Adolescents: Are There Any Gender Differences?
Source: Front Psychiatry. 2022 Jun 20;13:894218. doi: 10.3389/fpsyt.2022.894218 (PMC9251494; doi:10.3389/fpsyt.2022.894218)
Supplement: Supplementary file 1 [file Data_Sheet_1.pdf]

Supplementary Table 1. The prevalence of NSSI by psychological symptoms

Variable	Total		Boys		Girls	
	n(%)	*p*-value	n(%)	*p*-value	n(%)	*p*-value
**Psychological symptoms**						
No	2139(19.0)	<0.001	1224(21.3)	<0.001	915(16.6)	<0.001
Yes	1733(48.5)		837(51.0)		896(46.4)	
Emotional symptoms						
No	1886(17.9)	<0.001	1118(20.6)	<0.001	768(15.1)	<0.001
Yes	1986(46.1)		943(48.3)		1043(44.3)	
Conduct symptoms						
No	1891(18.0)	<0.001	1066(20.1)	<0.001	825(15.9)	<0.001
Yes	1981(45.7)		995(47.7)		986(43.9)	
Social adaptation symptoms						
No	2330(20.1)	<0.001	1297(22.3)	<0.001	1033(17.9)	<0.001
Yes	1542(47.7)		764(49.9)		778(46.5)	

Abbreviations: NSSI=non-suicidal self-injury

10.3389/fpsyt.2022.894218

Supplementary Table 2. Number, % and adjusted OR of SA by psychological symptoms and NSSI

Variable	Total			Boys			Girls		
	n(%)	*OR(95%CI)* [a]	*p*-value	n(%)	*OR(95%CI)* [b]	*p*-value	n(%)	*OR(95%CI)* [b]	*p*-value
**Psychological symptoms**									
No	287(2.6)	1.0		114(2.0)	1.0		173(3.1)	1.0	
Yes	362(10.1)	3.41(2.88-4.04)	<0.001	145(8.8)	3.90(2.99-5.09)	<0.001	217(11.2)	3.15(2.52-3.93)	<0.001
Emotional symptoms									
No	245(2.3)	1.0		102(1.9)	1.0		143(2.8)	1.0	
Yes	404(9.4)	3.49(2.94-4.14)	<0.001	157(8.0)	3.80(2.91-4.97)	<0.001	247(10.5)	3.35(2.67-4.19)	<0.001
Conduct symptoms									
No	241(2.3)	1.0		92(1.7)	1.0		149(2.9)	1.0	
Yes	408(9.4)	3.43(2.89-4.07)	<0.001	167(8.0)	3.94(3.00-5.16)	<0.001	241(10.7)	3.14(2.51-3.92)	<0.001
Social adaptation symptoms									
No	330(2.8)	1.0		130(2.2)	1.0		200(3.5)	1.0	
Yes	319(9.9)	3.02(2.55-3.58)	<0.001	129(8.3)	3.24(2.48-4.22)	<0.001	190(11.4)	2.94(2.36-3.67)	<0.001
**NSSI**									
No	253(2.3)	1.0		108(2.0)	1.0		145(2.6)	1.0	
Yes	396(10.2)	4.31(3.63-5.11)	<0.001	151(7.3)	3.42(2.63-4.46)	<0.001	245(13.5)	5.04(4.02-6.32)	<0.001

Abbreviations: SA=suicidal attempt; NSSI=non-suicidal self-injury

[a] Adjusted for gender, age, regional areas, school, urban/rurality, mother’s education level, economic status of family and ACEs

[b] Adjusted for age, regional areas, school, urban/rurality, mother’s education level, economic status of family and ACEs
